# The Interplay of Mitochondrial Oxidative Stress and Endoplasmic Reticulum Stress in Cardiovascular Fibrosis in Obese Rats

**DOI:** 10.3390/antiox10081274

**Published:** 2021-08-11

**Authors:** Francisco V. Souza-Neto, Sara Jiménez-González, Beatriz Delgado-Valero, Raquel Jurado-López, Marie Genty, Ana Romero-Miranda, Cristina Rodríguez, María Luisa Nieto, Ernesto Martínez-Martínez, Victoria Cachofeiro

**Affiliations:** 1 Departamento de Fisiología, Facultad de Medicina, Instituto de Investigación Sanitaria Gregorio Marañón (IiSGM), Universidad Complutense de Madrid, 28040 Madrid, Spain; franvasc@ucm.es (F.V.S.-N.); saraji02@ucm.es (S.J.-G.); beadel02@ucm.es (B.D.-V.); rajurado@ucm.es (R.J.-L.); marie.genty@inserm.fr (M.G.); anarom12@ucm.es (A.R.-M.); 2 Institut de Recerca del Hospital de la Santa Creu i Sant Pau, 08025 Barcelona, Spain; crodriguezs@santpau.cat; 3 Instituto de Investigación Biomédica Sant Pau (IB Sant Pau), 08025 Barcelona, Spain; 4 Ciber de Enfermedades Cardiovasculares (CIBERCV), Instituto de Salud Carlos III, 28220 Majadahonda, Spain; ml.nieto@csic.es; 5 Instituto de Biología y Genética Molecular, CSIC-Universidad de Valladolid, 47002 Valladolid, Spain

**Keywords:** cardiovascular fibrosis, endoplasmic reticulum stress, mitochondrial oxidative stress, obesity

## Abstract

We have evaluated the role of mitochondrial oxidative stress and its association with endoplasmic reticulum (ER) stress activation in the progression of obesity-related cardiovascular fibrosis. MitoQ (200 µM) was orally administered for 7 weeks to male Wistar rats that were fed a high-fat diet (HFD, 35% fat) or a control diet (CT, 3.5% fat). Obese animals presented cardiovascular fibrosis accompanied by increased levels of extracellular matrix proteins and profibrotic mediators. These alterations were associated with ER stress activation characterized by enhanced levels (in heart and aorta vs. CT group, respectively) of immunoglobulin binding protein (BiP; 2.1-and 2.6-fold, respectively), protein disulfide-isomerase A6 (PDIA6; 1.9-fold) and CCAAT-enhancer-binding homologous protein (CHOP; 1.5- and 1.8-fold, respectively). MitoQ treatment was able to prevent (*p* < 0.05) these modifications at cardiac and aortic levels. MitoQ (5 nM) and the ER stress inhibitor, 4-phenyl butyric acid (4 µM), were able to block the prooxidant and profibrotic effects of angiotensin II (Ang II, 10^−6^ M) in cardiac and vascular cells. Therefore, the data show a crosstalk between mitochondrial oxidative stress and ER stress activation, which mediates the development of cardiovascular fibrosis in the context of obesity and in which Ang II can play a relevant role.

## 1. Introduction

Cardiovascular fibrosis is a common feature in the context of obesity that occurs due to the imbalance between production and degradation of extracellular matrix components (ECM), mainly collagen type I [1]. This excessive ECM deposit due to an increased number of myofibroblasts, the main cell responsible for fibrosis, can produce an aberrant remodeling that triggers functional alterations by reducing the relaxing capability of the heart, which can in turn increase its filling pressure and contribute to diastolic dysfunction [2]. Vascular fibrosis is associated with arterial stiffness, which is a main determinant of cardiovascular mortality [3].

A variety of factors has been suggested as potential mediators of cardiac fibrosis in the context of obesity, including oxidative stress [4,5,6]. Mitochondria are the main source of reactive oxygen species (ROS) in the cell through oxidative phosphorylation (OXPHOS) during the process of ATP synthesis [7]. In normal conditions, ROS levels in the mitochondria are balanced by an effective antioxidant defense. However, an excessive production of ROS can affect mitochondrial function and could lead to functional alterations of the heart [8,9]. In this line, some data have reported the relevance of mitochondrial oxidative stress in the development not only of cardiac fibrosis but also in the metabolic alterations in diet-induced obese rats [10,11]. In our previous study [12], we observed that obese animals presented an increase in body weight, as well as in adiposity index, which was associated with adipose tissue fibrosis and metabolic alterations. At cardiac level, obese animals exhibited hypertrophy, interstitial fibrosis and an increase in superoxide anion levels, as compared to the reference group. These modifications were prevented by treatment with the mitochondrial antioxidant, MitoQ, and were in absence of alterations in cardiac function or in blood pressure [9]. However, the mechanisms through which mitochondrial ROS plays a role in the fibrotic consequences of obesity remain unclear. In the last years, it has been described that obesogenic diets have an impact on the diversity and functionality of the gut microbiota, which plays a central role in the cardiovascular alterations observed in obesity [13,14]. In this sense, alterations in gut microbiota are associated with renal disorders [15] and cardiovascular diseases such as heart failure [16], atherosclerosis [17], hypertension [18] or vascular dysfunction [19]. Systemic inflammation plays a central role in all of these pathologies [20,21]. It has been observed that changes in gut microbiota observed in obese animals are involved in adipose tissue inflammation, oxidative stress and metabolic disorders [22]. In a recent study, we have demonstrated the relevance of oxidative stress in all of these alterations, since treatment with a mitochondrial antioxidant was able to prevent insulin resistance and cardiac fibrosis observed in obese animals. These beneficial effects of the mitochondrial antioxidant were accompanied by a restoration in gut microbiota composition [23], thus showing the link between microbiota dysbiosis, oxidative stress, inflammation and cardiac alterations in the context of obesity. In this study, we would like to go deeper into the cardiac fibrosis associated with obesity, as well as the role of mitochondrial oxidative stress.

The endoplasmic reticulum (ER) is a dynamic cellular organelle with different functions: Ca^2+^ buffering, lipid and carbohydrate metabolism, as well as protein synthesis, folding and processing, among others. In a physiological state, protein synthesis and folding levels follow a balance so that the folding capacity of the ER is not saturated. However, this balance is perturbed in response to different stimuli, including oxidative stress, and misfolded proteins thus accumulate in the ER lumen. This promotes ER stress and the activation of the unfolded protein response (UPR) to normalize and return the balance in which binding immunoglobulin protein (BiP) plays a central role as a chaperone. BiP is therefore considered to be a marker of ER stress activation. Different studies have demonstrated the participation of ER stress in cardiovascular damage. The contribution of ER stress to cardiac hypertrophy has been demonstrated in transgenic mice, where the genetic ablation of mediators of ER stress reduced cardiac remodeling, as well as cardiac dysfunction, after transverse aortic constriction in mice [24]. In addition, the beneficial effects of ER stress inhibition were confirmed by the pharmacological inhibition of ER stress capability to reduce cardiac hypertrophy induced by a pressure overload model [25]. Ischemic conditions are also associated with impaired protein folding and subsequent activation of ER stress. In prolonged ER stress conditions, ER activates proapoptotic signaling pathways, thereby contributing to the onset of heart failure [26]. At vascular level, preclinical and clinical studies have elucidated the activation of ER stress in atherosclerotic plaques. The apoptosis in vascular smooth muscle cells (VSMCs) derived from prolonged ER stress activation leads to plaque rupture, oxidative stress and enhanced calcification [27,28]. In addition, the role of ER stress has been demonstrated in vascular endothelial dysfunction in hypertension [29]. Different studies have reported the potential participation of ER stress in the development of cardiac fibrosis in obese models [30,31].

The mitochondria and ER can come into contact at different sites, forming the mitochondria–ER associated membranes, through which they can coordinate their functions by participating in the regulation of different processes [32]. However, whether these interactions can be relevant for the development of cardiovascular fibrosis in the context of obesity is not fully established. Therefore, the aim of this study was to evaluate whether the participation of oxidative stress in the development of cardiovascular fibrosis in the context of obesity is through the activation of ER stress. To address this aim, we evaluated the effect of the mitochondria-targeted antioxidant MitoQ in cardiovascular fibrosis in obese rats. In addition, we have studied how MitoQ treatment can affect ER stress activation. We have also analyzed the crosstalk between both processes in cultured cardiac fibroblasts and VSMCs, the main cells involved in ECM synthesis.

## 2. Materials and Methods

### 2.1. Animal Model

Male Wistar rats of 150 g (6 weeks old; Envigo, Barcelona, Spain) were fed either a high-fat diet (HFD, 35% fat; Envigo Teklad no. TD.03307, Haslett, MI, USA; *n* = 16) or a standard diet (CT, 3.5% fat; Envigo Teklad no.TD.2014, Haslett, MI, USA; *n* = 16) for a period of 7 weeks. The mitochondrial antioxidant MitoQ (200 µM) was administered to half of the animals of each group in the drinking water for the same period of time. Animals were sacrificed by decapitation. The dose of MitoQ was based on previous data [33]. MP Murphy from medical Research Council Mitochondrial Biology Unit (Biomedical Campus, Cambridge, UK) provided MitoQ. Animals were weighed once a week. All experimental procedures were approved by the Animal Care and Use Committee of Universidad Complutense de Madrid following the Spanish Policy for Animal Protection RD53/2013 and according the European Union Directive 2010/63/UE. Serum and plasma samples were collected at the end of the study.

### 2.2. Histological Analysis

Samples of aorta and heart were dehydrated, immersed in paraffin and cut into sections 4 µm thick. Collagen fibers were detected by the staining of sections with picrosirius red. Aorta and coronary artery fibrosis was quantified in the media layer as the ratio of collagen deposition or interstitial fibrosis to the total media area. Ten to fifteen fields for each sample were analyzed with an objective (40×) under microscopy of transmitted light (Leica DM 2000; Leica AG, Wetzlar, Germany). Quantification of the data was performed with an analysis system (Leica LAS 4.3; Leica AG, Wetzlar, Germany).

### 2.3. Cell Isolation and Cell Culture Conditions

Heart and thoracic aorta of adult male Wistar rats (weighing 250–300 g) were used to isolate cardiofibroblasts and VSMCs. Differential centrifugation was performed after enzymatic digestion of the hearts with a mix of collagenase-trypsin for the isolation of cardiac fibroblasts and aorta with a mix of collagenase-elastase for the isolation of VSMCs, as previously described [5,34]. Cells were used between passages 5 and 6. DMEM medium supplemented with 10% FBS, 10 mmol/L L-glutamine, 100 U/mL penicillin/streptomycin, 10 mmol/L L-pyruvate and 2 mmol/L HEPES was used for cardiac fibroblasts. VSMCs were maintained in DMEM F-12 supplemented with 10% FBS and 100 U/mL penicillin/streptomycin. Cells were grown as monolayer culture in a T-75 tissue culture flask from an initial density of 0.5 × 10^6^ cells. Confluent cells were passaged with 0.25% trypsin in 0.01% EDTA. Cells were serum-starved for 12 h, and 6-well plates were used with 90% confluence for each experimental condition. All assays in the present study were done at temperatures of 37 °C, 95% sterile air and 5% CO_2_ in a saturation humidified incubator. Cells were treated with angiotensin II (Ang II; 10^−6^ M) for 24 h in the presence or absence of the mitochondrial antioxidant, MitoQ (5 nM), or the chemical chaperone, 4-phenyl butyric acid (4-PBA; 4µM). VSMCs were treated with the ER stress inducer, tunicamycin (1–2 µg/mL), for 24 h.

### 2.4. Western Blot

Total proteins from cardiac and aortic homogenates, as well as cellular extracts, were prepared as previously described [9]. Briefly, proteins were separated by SDS-PAGE on polyacrylamide Criterion^TM^ TGX^TM^ Precast Gels (BioRad, Hercules, CA, USA) and transferred to Hybond-c Extra nitrocellulose membranes (Hybond-P; Amersham Biosciences, Piscataway, NJ, USA). Membranes were reused after stripping a maximum of 3 times. This allows for measuring the greatest number of proteins in the same membrane. Membranes were probed with primary antibodies for activating transcription factor 6-alpha (ATF6α; Santa Cruz, TX, USA; dilution 1:250), immunoglobulin binding protein (BiP; BD Biosciences, Madrid, Spain; dilution 1:1500), CCAAT-enhancer-binding homologous protein (CHOP; Cell Signaling Technology, Danvers, MA, USA; dilution 1:500), collagen I (Calbiochem, San Diego, CA, USA; dilution 1:500), connective tissue growth factor (CTGF; Sigma-Aldrich, San Louis, MO, USA; dilution 1:1000), protein disulfide-isomerase A6 (PDIA6; Abcam plc, Cambridge, UK; dilution 1:500), transforming growth factor-β (TGF-β; Abcam plc, Cambridge, UK; dilution 1:250) and α-tubulin or β-actin (Sigma-Aldrich, San Louis, MO, USA; dilution 1:5000) as loading controls. Signals were detected using the ECL system (Millipore, Burlington, MA, USA). Results are expressed as an *n*-fold increase over the values of the control group in densitometric arbitrary units.

### 2.5. Retrotranscription and Real-Time PCR

Total RNA was isolated using Trizol Reagent (Fisher Scientific, Waltham, MA, USA) and was reverse-transcribed into cDNA using the High-Capacity cDNA Reverse Transcription Kit (Thermo Fisher Scientific Inc, Waltham, MA, USA). Quantitative PCR analysis was performed with SYBR green PCR technology (Bio-Rad, Hercules, CA, USA) and specific oligonucleotides (Table S1). Quantification of mRNA levels was performed by real-time PCR using the ΔΔCt method. Data were normalized to hypoxanthine phosphoribosyltransferase (HPRT).

### 2.6. Measurement of Intracellular Superoxide Anion Production

Cardiomyofibroblasts and VSMCs were seeded into 48-well plates at 70% confluence for experiments measuring O_2_^−^ production. Cells were exposed for 24 h with either vehicle or Ang II (10^−6^ M) for 24 h in the presence or absence of the mitochondrial antioxidant, MitoQ (5 nM), or the chemical chaperone, 4-PBA (4 µM). Cells were then exposed with dihydroethidium (DHE; 5 × 10^−3^ mmol/L) for 30 min. Cells were washed with warm PBS and analyzed with an objective lens (40×) in a fluorescent laser scanning Leica DMI 3000 microscope. A number of cells (150–200) for each condition was analyzed. The mean fluorescence densities in the nucleus were calculated. Results are expressed as an *n*-fold increase over the data of the control group. The analysis was performed by a single researcher unaware of the experimental groups.

### 2.7. Statistical Analysis

Variables are expressed as mean ± SEM. Kolmogorov–Smirnov test was used to verify the normality of distributions. Differences between 2 groups were assessed with Student’s t test. Differences among several groups were evaluated using one-way ANOVA. Newman–Keuls test was used as a post hoc test for differences in means. A value of *p* < 0.05 was used as the cutoff for defining statistical significance. Data were analyzed using the statistical program GraphPad Prism 5 (San Diego, CA, USA).

## 3. Results

### 3.1. Mitochondrial Oxidative Stress Promotes ECM Protein Deposition and ER Stress Activation at Cardiac Level in Obesity

**Figure 1:**
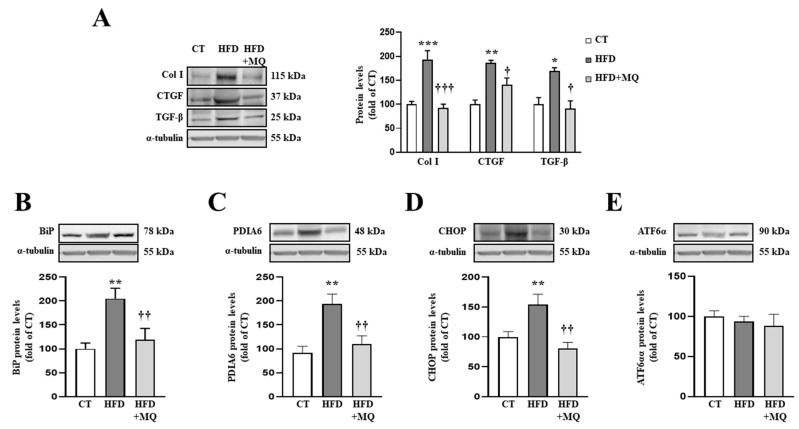
Mitochondrial oxidative stress mediates the increase in extracellular matrix proteins and endoplasmic reticulum stress at cardiac level. Protein levels of (**A**) collagen type I (Col I), connective tissue growth factor (CTGF) and transforming growth factor-beta (TGF-β); (**B**) immunoglobulin binding protein (BiP); (**C**) protein disulfide isomerase family A member 6 (PDIA6); (**D**) CCAAT-enhancer-binding protein homologous protein (CHOP); (**E**) activating transcription factor 6-alpha (ATF6α) in heart tissue from control rats fed a normal chow (CT) and rats fed a high-fat diet (HFD) treated with vehicle or with the mitochondrial antioxidant MitoQ (MQ; 200 µM). Bars graphs represent the mean ± SEM of 6–8 animals normalized for α-tubulin. * *p* < 0.05, ** *p* < 0.01, *** *p* < 0.001 vs. control group. † *p* < 0.05, †† *p* < 0.01, ††† *p* < 0.001 vs. HFD group.

HFD animals presented an increase in collagen type I protein levels accompanied by an increase in the profibrotic mediators CTGF and TGF-β as compared to control animals (Figure 1A). The treatment with the mitochondrial antioxidant, MitoQ, was able to prevent the increase in ECM components and profibrotic growth factors observed in HFD (Figure 1A).

In order to identify possible mechanisms involved in the development of cardiac alterations in HFD animals and the improvement showed by MitoQ in ECM production, we evaluated ER stress activation. Obese animals presented an increase in two ER stress markers, such as BiP and PDIA6 (Figure 1B,C, respectively). Complementary analyses of different pathways involved in ER stress activation revealed an increase in CHOP protein levels without modifications in ATF6α protein levels (Figure 1D,E, respectively). MitoQ treatment was able to prevent all of these alterations, demonstrating the crosstalk between mitochondria and ER (Figure 1B–D). Interestingly, ER stress markers BiP and PDIA6 (Figure S1A,B) correlated with cardiac interstitial fibrosis in the animals, as well as CHOP correlating with collagen type I protein levels (Figure S1C), thereby showing the possible role of ER stress in ECM production.

### 3.2. MitoQ Treatment Improves Vascular Fibrosis and ER Stress Activation in the Aorta of HFD Animals

**Figure 2:**
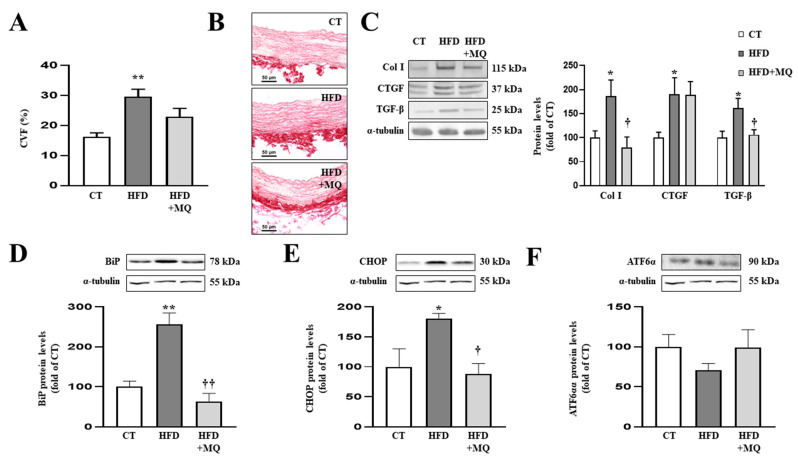
Mitochondrial oxidative stress mediates vascular fibrosis and endoplasmic reticulum stress at aortic level. (**A**) Quantification of collagen volume fraction and (**B**) representative microphotographs of aortic sections staining with picrosirius red. Protein levels of (**C**) collagen type I (Col I), connective tissue growth factor (CTGF) and transforming growth factor-beta (TGF-β); (**D**) immunoglobulin binding protein (BiP); (**E**) CCAAT-enhancer-binding protein homologous protein (CHOP); (**F**) activating transcription factor 6-alpha (ATF6α) in aortic tissue from control rats fed a normal chow (CT) and rats fed a high-fat diet (HFD) treated with vehicle or with the mitochondrial antioxidant MitoQ (MQ; 200 µM). Scale bar: 50 µm. Bars graphs represent the mean ± SEM of 6–8 animals normalized for α-tubulin. * *p* < 0.05, ** *p* < 0.01 vs. control group. † *p* < 0.05, †† *p* < 0.01 vs. HFD group.

Having observed the effects of obesity and participation of mitochondrial oxidative stress in cardiac alterations, we examined whether the inhibition of mitochondrial oxidative stress could improve vascular fibrosis in HFD animals. Diet-induced obese animals showed important modifications at vascular level, characterized by aortic fibrosis (Figure 2A,B) accompanied by collagen type I and the upregulation of profibrotic mediators CTGF and TGF-β (Figure 2C). In addition, obese animals presented an increase in fibrosis in the media of the descending coronary artery, which was partially decreased by MitoQ treatment (Figure S2A,B). As occurs with cardiac interstitial fibrosis, ER stress markers correlated with fibrosis observed in the media of the coronary vessel (Figure S2C,D). All of these modifications observed in obese animals were in the absence of alterations in aortic morphology, such as those of the media or lumen area (Figure S3A–C). Mitochondrial oxidative stress participates in vascular fibrosis, since treatment with MitoQ was able to prevent aortic fibrosis and increases in collagen type I and TGF-β protein levels without modifications in CTGF expression (Figure 2A–C).

As occurs at cardiac level, obese animals presented aortic ER stress activation characterized by an increase in BiP protein levels (Figure 2D) together with CHOP upregulation (Figure 2E), while ATF6α protein levels remained unchanged (Figure 2F) compared to control rats. Treatment with MitoQ was able to prevent ER stress activation as well as increases in CHOP protein levels in obese animals (Figure 2D,E).

### 3.3. Mitochondrial Oxidative Stress Mediates the Effects of Ang II in Cardiac Fibroblasts

**Figure 3:**
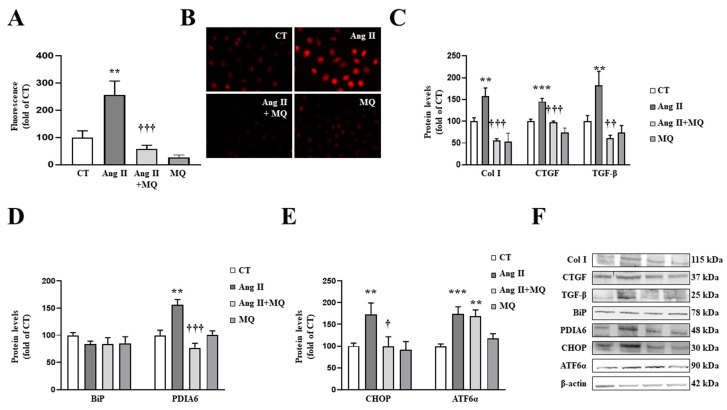
Mitochondrial oxidative stress mediates the prooxidant, profibrotic and the endoplasmic reticulum stress activation induced by angiotensin II (Ang II) in cardiac fibroblasts. (**A**,**B**) Effect of the mitochondrial antioxidant MitoQ (MQ; 5 nM) on superoxide anion production. Quantification of cells labelled with the oxidative dye dihydroethidium (**A**) and representative microphotographs (magnification 40×) (**B**). Protein levels of (**C**) collagen type I (Col I), connective tissue growth factor (CTGF) and transforming growth factor-beta (TGF-β); (**D**) immunoglobulin binding protein (BiP) and protein disulfide isomerase family A member 6 (PDIA6); (**E**) CCAAT-enhancer-binding protein homologous protein (CHOP) and activating transcription factor 6-alpha (ATF6α) in cardiac fibroblasts treated with Ang II (10^−6^ M) for 24 h in the presence or in the absence of MQ. (**F**) Representative blots for protein expressions. Bars graphs represent the mean ± SEM of MS four to six assays normalized for β-actin. ** *p* < 0.01, *** *p* < 0.001 vs. control cells. † *p* < 0.05, †† *p* < 0.01, ††† *p* < 0.001 vs. Ang II-treated cardiac fibroblasts.

Obese animals presented an increase in Ang II plasma levels, which was prevented by MitoQ treatment (Figure S4). In order to study the direct effects of mitochondrial oxidative stress on ECM production and on Ang II effects, we exposed cardiac fibroblasts to Ang II in presence or absence of the mitochondrial antioxidant MitoQ. Ang II (10^−6^ M) was able to increase superoxide anion production levels in cardiac fibroblasts (Figure 3A,B). As expected, Ang II-treated cells presented a significant increase in collagen type I protein levels, as well as levels of CTGF and TGF-β (Figure 3C). MitoQ (5 nM) prevented the prooxidant effects of Ang II, showing the efficiency of the treatment (Figure 3A,B). In addition, MitoQ treatment blunted the profibrotic effects of Ang II, thus preventing an increase in collagen I, CTGF and TGF-β protein levels in cardiac fibroblasts (Figure 3C).

Regarding ER stress, Ang II-treated cardiac cells increased a marker of ER stress activation, such as PDIA6, without modifications in BiP protein levels at 24 h (Figure 3D). The activation of ER stress was confirmed, since Ang II promoted an upregulation of CHOP and ATF6α protein levels in cardiac fibroblasts (Figure 3E). The presence of MitoQ in the culture medium was able to prevent the increase in PDIA6 and CHOP induced by Ang II; however, it was not able to modify the increase in ATF6α protein levels in Ang II-treated cells (Figure 3D,E).

### 3.4. Mitochondrial Oxidative Stress Mediates the Effects of Ang II in Vascular Smooth Muscle Cells

Having observed the beneficial effects of MitoQ at vascular level in obese animals and the effects of Ang II on cardiac fibroblasts, we treated VSMCs with Ang II and MitoQ.

**Figure 4:**
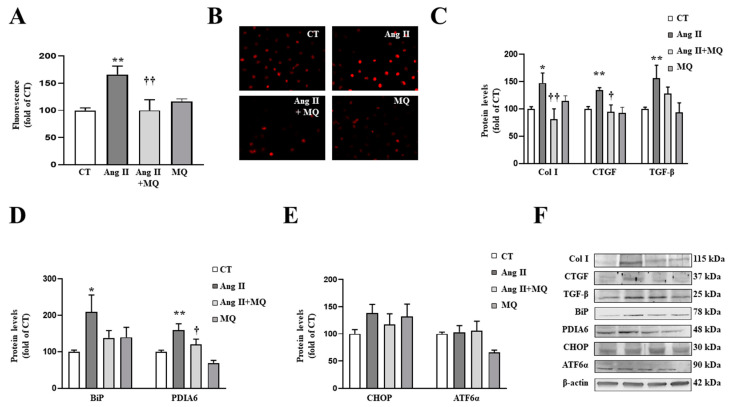
Mitochondrial oxidative stress mediates the prooxidant, profibrotic and the endoplasmic reticulum stress activation induced by angiotensin II (Ang II) in vascular smooth muscle cells (VSMCs). (**A**,**B**) Effects of the mitochondrial antioxidant MitoQ (MQ; 5 nM) on superoxide anion production. Quantification of cells labelled with the oxidative dye dihydroethidium (**A**) and representative microphotographs (magnification 40×) (**B**). Protein levels of (**C**) collagen type I (Col I), connective tissue growth factor (CTGF) and transforming growth factor-beta (TGF-β); (**D**) immunoglobulin binding protein (BiP) and protein disulfide isomerase family A member 6 (PDIA6); (**E**) CCAAT-enhancer-binding protein homologous protein (CHOP) and activating transcription factor 6-alpha (ATF6α) in VSMCs treated with Ang II (10^−6^ M) for 24 h in the presence or in the absence of MQ. (**F**) Representative blots for protein expressions. Bars graphs represent the mean ± SEM of four to six assays normalized for β-actin. * *p* < 0.05, ** *p* < 0.01, vs. control cells. † *p* < 0.05, †† *p* < 0.01, vs. Ang II-treated VSMCs.

The presence of MitoQ (5 nM) was able to block the prooxidant and profibrotic effects of Ang II (10^−6^ M) in VSMCs, since it was able to prevent the increase in superoxide anion production (Figure 4A,B), as well as the upregulation in collagen type I, CTGF and TGF-β protein levels (Figure 4C) induced by Ang II.

As occurs in cardiac cells, Ang II activated ER stress characterized by an increase in BiP and PDIA6 protein levels (Figure 4D). Analysis of pathways involved in ER stress activation showed that Ang II was not able to produce a significant increase in CHOP or ATF6α after 24 h of stimulation (Figure 4E). The effects of Ang II on BiP and PDIA6 protein levels were blunted by the presence of MitoQ in the culture medium (Figure 4D).

### 3.5. Inhibition of Endoplasmic Reticulum Stress Blocks the Prooxidant and Profibrotic Effects of Ang II in Cardiovascular Cells

**Figure 5:**
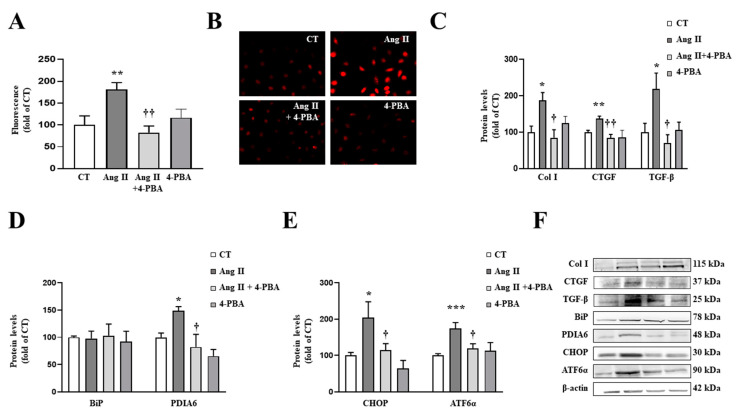
Endoplasmic reticulum stress mediates the prooxidant and profibrotic effects induced by angiotensin II (Ang II) in cardiac fibroblasts. (**A**,**B**) Effect of 4-phenylbutyrate acid (4-PBA; 4 µM) on superoxide anion production. Quantification of cells labelled with the oxidative dye dihydroethidium (**A**) and representative microphotographs (magnification 40×) (**B**). Protein levels of (**C**) collagen type I (Col I), connective tissue growth factor (CTGF) and transforming growth factor-beta (TGF-β); (**D**) immunoglobulin binding protein (BiP) and protein disulfide isomerase family A member 6 (PDIA6); (**E**) CCAAT-enhancer-binding protein homologous protein (CHOP) and activating transcription factor 6-alpha (ATF6α) in cardiac fibroblasts treated with Ang II (10^−6^ M) for 24 h in the presence or in the absence of 4-PBA. (**F**) Representative blots for protein expressions. Bars graphs represent the mean ± SEM of four to six assays normalized for β-actin. * *p* < 0.05, ** *p* < 0.01, *** *p* < 0.001 vs. control cells. † *p* < 0.05, †† *p* < 0.01 vs. Ang II-treated cardiac fibroblasts.

In order to explore the direct effects of ER stress activation on Ang II actions, cardiac fibroblasts were treated with Ang II in the presence or absence of the ER stress inhibitor, 4-PBA. The presence of 4-PBA (4 µM) was able to prevent the increase in superoxide anion production in Ang II-treated cells (Figure 5A,B), thereby demonstrating a vicious circle between oxidative stress and ER stress. In addition, the pharmacological inhibition of ER stress prevented the profibrotic actions of Ang II (10^−6^ M) by decreasing the up-regulation of collagen type I and the profibrotic mediators CTGF and TGF-β protein levels in cardiac fibroblasts (Figure 5C).

As expected, the presence of 4-PBA was able to inhibit ER stress activation in cardiac fibroblasts treated with Ang II, characterized by a decrease in PDIA6 (Figure 5D) protein levels. In addition, 4-PBA was able to prevent the increase in CHOP and ATF6α protein levels induced by Ang II (Figure 5E).

**Figure 6:**
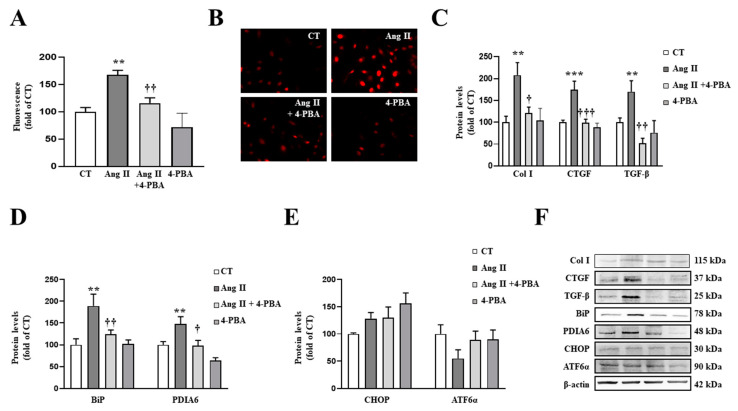
Endoplasmic reticulum stress mediates the prooxidant and profibrotic effects induced by Angiotensin II (Ang II) in vascular smooth muscle cells (VSMCs). (**A**,**B**) Effect of 4-phenylbutyrate acid (4-PBA; 4 µM) on superoxide anion production. Quantification of cells labelled with the oxidative dye dihydroethidium (**A**) and representative microphotographs (magnification 40×) (**B**). Protein levels of (**C**) collagen type I (Col I), connective tissue growth factor (CTGF) and transforming growth factor-beta (TGF-β); (**D**) immunoglobulin binding protein (BiP) and protein disulfide isomerase family A member 6 (PDIA6); (**E**) CCAAT-enhancer-binding protein homologous protein (CHOP) and activating transcription factor 6-alpha (ATF6α) in VSMCs treated with Ang II (10^−6^ M) for 24 h. (**F**) Representative blots for protein expressions. Bars graphs represent the mean ± SEM of four to six assays normalized for β-actin. ** *p* < 0.01, *** *p* < 0.001 vs. control cells. † *p* < 0.05, †† *p* < 0.01, ††† *p* < 0.001 vs. Ang II-treated VSMCs.

Similar results were obtained in VSMCs. The presence of 4-PBA (4 µM) blunted the prooxidant (Figure 6A,B) and the profibrotic (Figure 6C) actions of Ang II (10^−6^ M) in VSMCs. These effects of 4-PBA were accompanied by the inhibition of BiP and PDIA6 up-regulation in Ang II-treated cells, thereby confirming the effectiveness of the treatment (Figure 6D).

By complementary analyses, we confirmed the direct effects of ER stress in VSMCs through the use of tunicamycin, a pharmacological inducer. Tunicamycin promoted an increase in superoxide anion production in a dose-dependent manner (Figure S5A). At 1.5 µg/mL, tunicamycin induced an increase in collagen type I mRNA levels, an effect that was accompanied by TGF-β mRNA level up-regulation in VSMCs (Figure S5B,C).

## 4. Discussion

The purpose of this study was to investigate the interactions between mitochondrial oxidative stress and ER stress in cardiovascular fibrosis associated with obesity. In the present study, ER stress, which is activated in the cardiovascular system in obesity, emerges as a potential mediator of cardiovascular fibrosis induced by mitochondrial oxidative stress in this context. This is based on the fact that the reduction in mitochondrial oxidative stress by MitoQ prevented the upregulation not only of total collagen but also of collagen I and the profibrotic mediators TGF-β and CTGF protein levels in obese rats. These effects were accompanied by an attenuation of ER stress at cardiac and vascular levels. In addition, ROS reduction and ER stress inhibition by MitoQ or 4-PBA, respectively, reduced the profibrotic actions of Ang II, whose levels are elevated in obese animals and involved in cardiovascular fibrosis associated with obesity in cardiac and vascular cells [35,36]. This suggests that interactions between ER stress and mitochondrial oxidative stress regulate downstream events and are responsible for fibrosis in the context of obesity. Thus, ER stress emerges as a potential therapeutic target involved in the altered ECM processing observed in obesity.

Fibrosis is a common feature involved in several cardiovascular pathologies such as obesity, hypertension, myocardial infarction and atherosclerosis, among others [23,37,38]. Cardiovascular fibrosis contributes to poor prognosis in obesity due to alterations in the cardiac and vascular architecture, thus favoring vascular stiffness, cardiac dysfunction and heart failure [39,40]. However, the signaling pathways involved in this process are not fully understood. Therefore, it is mandatory to comprehend the pathological context in order to identify new possible therapeutic approaches. Several studies have elucidated the main role of inflammation in cardiac damage, thereby promoting cardiac dysfunction and myocardial necrosis [41]. It has been described that proinflammatory cytokines such as tumor necrosis-alpha (TNF-α), interleukin (IL)-1β and IL-6 play a central role in cardiac damage associated with ischemic conditions [42] and obesity, as well [43]. In a recent study, Zhou J et al. demonstrated that pretreatment with miR-181a-5p antagomiR was able to improve cardiac function in an animal model of cardiac damage. This effect was accompanied by an improvement in both oxidative stress and inflammation of the cardiac tissue [44]. At vascular level, it is known that inflammation and oxidative stress contribute to endothelial dysfunction, as has been recently reported in clinical studies [45]. In addition, inflammation and oxidative stress are involved in the initial phase of cardiac remodeling and facilitate ECM deposition and subsequent fibrosis. Our present study extends the previous observation made at the cardiac level [9] to the vascular level, since treatment with the mitochondrial antioxidant MitoQ was able to reduce total collagen in the media of the descending coronary artery and in the aorta of obese rats. Thus, this supports the role of mitochondrial oxidative stress in the cardiovascular fibrosis in obesity, which confirms previous observations [46]. The amelioration in cardiovascular fibrosis was accompanied by a reduction in collagen type I protein levels in MitoQ-treated animals. Collagen type I is the predominant collagen subtype found in the arterial wall (70–75%) [47] and within the heart (approximately 80%). TGF-β and CTGF are two profibrotic mediators involved in collagen synthesis and subsequent fibrosis [48,49]. In this line, MitoQ was able to prevent the increase in TGF-β and CTGF at cardiac and vascular levels (except for CTGF aortic protein levels) in the animals fed an HFD, suggesting that the antifibrotic effects of MitoQ are related to collagen synthesis. This improvement could also be related to the interactions between gut microbiota and mitochondrial oxidative stress, which participates in cardiac fibrosis in the context of obesity [23]. In fact, the role of microbiota dysbiosis has been reported not only in obesity but also in other pathological scenarios, including neurodegenerative diseases [50,51,52,53,54].

Interestingly, treatment with the mitochondrial antioxidant was also able to prevent cardiovascular ER stress activation in obese animals. HFD rats presented an increase in BiP and PDIA6, another ER stress marker, at cardiac protein levels, which was prevented by MitoQ treatment. The effect of MitoQ on ER stress was confirmed at vascular level, since MitoQ was also able to prevent upregulation in BiP aortic protein levels observed in HFD animals, showing the connection between mitochondria and ER at cardiovascular level, as has been reported in other pathological conditions, including metabolic alterations [55,56,57,58].

The initial response of ER in restoring homeostasis is the UPR, which is initiated by three transmembrane proteins: inositol requiring 1 (IRE1), PKR-like ER kinase (PERK), and ATF6. Our data show that obese rats presented an increase in cardiac and aortic protein levels of CHOP, a common downstream element of IRE1 and PERK, without modifications in ATF6α protein levels. The upregulation of CHOP in obese rats was blunted by MitoQ treatment, confirming the crosstalk between mitochondrial oxidative stress and ER stress activation in obese animals. Similarly, minipigs fed an HFD presented cardiac interstitial fibrosis, which was accompanied by ER stress activation characterized by CHOP upregulation [30]. CHOP is a transcription factor that mediates apoptosis, triggered by UPR response, and has been highlighted as a key regulator of cardiac injury. The increase in CHOP levels could also be ascribed to the mitochondrial UPR (UPR^MT^); however, our results showing higher levels of BiP and PDIA6 reflect the activation of ER stress in obesity, in agreement with previous reports, while the contribution of UPR^MT^ to cardiovascular alterations in obesity is currently uncertain. Previous studies have demonstrated that genetic disruption of CHOP attenuates cardiac hypertrophy and cardiac dysfunction in mice induced by pressure overload [59]. Thus, this supports that ER stress activation could become a novel target for cardiovascular diseases. In addition, ER stress activation has been previously observed in adipose tissue of obese patients, and a reduction in body weight is associated with a decrease in ER stress markers [60]. Moreover, the administration of pharmacological inhibitors of ER stress was able to prevent UPR activation in liver and adipose tissue by improving insulin resistance in genetic models of obesity [61]. Therefore, these data support the role of ER activation in the metabolic alterations associated with obesity.

Different factors are involved in cardiovascular damage, including the renin–angiotensin system, which can enhance ROS generation in myocardial tissue [62]. It has been described that Ang II, a well-known profibrotic factor at cardiac and vascular levels [63,64], is elevated in obese patients [65]. In agreement with this, we have observed a similar increase in Ang II plasma levels in obese rats, which was normalized by MitoQ treatment. In cultured cardiac fibroblasts and VSMCs, Ang II increased superoxide anion production and promoted an increase in collagen type I protein levels accompanied by an up-regulation of TGF-β and CTGF expression. The presence of MitoQ was able to prevent not only the prooxidant effects of Ang II but also its profibrotic effects. This fact shows that mitochondrial oxidative stress mediates the profibrotic and prooxidant effects of Ang II and its involvement in ECM production in obesity, reinforcing the data observed in vivo. Importantly, cardiac and vascular cells exposed to Ang II presented ER stress activation characterized by an upregulation of ER stress markers. This effect was previously observed in adipocytes [66], in podocytes [67] and in pancreatic β cells [68]. We show for the first time that Ang II promotes ER stress activation in cardiac fibroblasts and VSMCs through mitochondrial oxidative stress production. However, it is important to mention that this ER stress activation seems to operate through different pathways in cardiac fibroblasts and VSMCs, since up-regulation in CHOP and ATF6α protein levels observed in the cardiac cells under Ang II treatment was not present in VSMCs.

A previous study has demonstrated that the pharmacological inhibition of ER stress is able to prevent cardiac fibrosis induced by Ang II infusion in mice [29]. In agreement with this, we have shown that treatment with the ER stress inhibitor, 4-PBA, prevents the increase in ECM proteins induced by Ang II in cardiac fibroblasts and VSMCs. The role of ER stress in ECM production was confirmed by the pharmacological inducer of ER stress, tunicamycin. VSMCs exposed to tunicamycin presented an increase in collagen type I and TGF-β mRNA levels. Moreover, ER stress activation by tunicamycin also promoted ROS production in VSMCs, thereby supporting the notion that there exists a crosstalk between oxidative stress and ER stress. ER is an organelle responsible for calcium homeostasis. It has been described that a massive influx of calcium into mitochondria can lead to the formation and opening of pores, thereby promoting damage to mitochondria and the subsequent ROS production [69,70]. In this sense, we have demonstrated that Ang II promotes superoxide anion production in cardiovascular cells, an effect that was prevented by ER stress inhibition. 

## 5. Conclusions

In summary, we have demonstrated that the cardiovascular fibrosis observed in obese animals was accompanied by oxidative stress and ER stress activation. Inhibition of mitochondrial oxidative stress with MitoQ prevented all of these alterations, thus supporting the role of ER stress in the profibrotic effect of mitochondrial oxidative stress in the context of obesity. Moreover, there seems to be a crosstalk between mitochondrial oxidative stress and ER stress activation, which mediates the increase in ECM proteins, ROS production and ER stress activation in Ang II-treated cardiovascular cells, a factor involved in the cardiovascular fibrosis associated with obesity [36,71].

These results show the potential role of mitochondrial oxidative stress and ER stress in cardiovascular fibrosis in the context of obesity, thereby suggesting new therapeutic approaches in the management of obesity for the treatment of cardiovascular alterations in which high levels of Ang II can play a role.

## 6. Limitations

Although the study explores interactions between mitochondrial oxidative stress and endoplasmic reticulum stress in cardiovascular fibrosis in obese rats through different approaches (in vivo and in vitro studies), the data do not allow the assessment that one ultimately leads to the other. The information regarding specific time course of events could represent a limitation of our study.

## Data Availability

The data used to support the findings of this study are available from the corresponding author upon request.

## Supplementary Materials

The following are available online at https://www.mdpi.com/article/10.3390/antiox10081274/s1. Table S1: Primers used in real time PCR analysis, Figure S1: Correlations observed between cardiac fibrosis and endoplasmic reticulum stress, Figure S2: Mitochondrial oxidative stress mediates the fibrosis of the media of the descending coronary artery, Figure S3: Vascular morphology of aorta in the animals, Figure S4: Angiotensin (Ang II) plasma levels, Figure S5: Endoplasmic reticulum stress increases oxidative stress and extracellular matrix markers in vascular smooth muscle cells.

## References

[1] Kong P., Christia P., Frangogiannis N.G. (2014). The pathogenesis of cardiac fibrosis. Cell Mol. Life Sci..

[2] Czubryt M.P. (2019). Cardiac Fibroblast to Myofibroblast Phenotype Conversion-An Unexploited Therapeutic Target. J. Cardiovasc. Dev. Dis..

[3] Kirkman D.L., Robinson A.T., Rossman M.J., Seals D.R., Edwards D.G. (2021). Mitochondrial contributions to vascular endothelial dysfunction, arterial stiffness, and cardiovascular diseases. Am. J. Physiol. Heart Circ. Physiol..

[4] Cavalera M., Wang J., Frangogiannis N.G. (2014). Obesity, metabolic dysfunction, and cardiac fibrosis: Pathophysiological pathways, molecular mechanisms, and therapeutic opportunities. Transl. Res..

[5] Martinez-Martinez E., Jurado-Lopez R., Valero-Munoz M., Bartolome M.V., Ballesteros S., Luaces M., Briones A.M., Lopez-Andres N., Miana M., Cachofeiro V. (2014). Leptin induces cardiac fibrosis through galectin-3, mTOR and oxidative stress: Potential role in obesity. J. Hypertens..

[6] Jeong J., Kim D.H., Park G., Park S., Kim H.S. (2019). Clinical significance of anti-dense fine speckled 70 antibody in patients with fibromyalgia. Korean J. Intern. Med..

[7] Bhatti J.S., Bhatti G.K., Reddy P.H. (2017). Mitochondrial dysfunction and oxidative stress in metabolic disorders—A step towards mitochondria based therapeutic strategies. Biochim. Biophys. Acta.

[8] Tsutsui H., Kinugawa S., Matsushima S. (2011). Oxidative stress and heart failure. Am. J. Physiol. Heart Circ. Physiol..

[9] Jimenez-Gonzalez S., Marin-Royo G., Jurado-Lopez R., Bartolome M.V., Romero-Miranda A., Luaces M., Islas F., Nieto M.L., Martinez-Martinez E., Cachofeiro V. (2020). The Crosstalk between Cardiac Lipotoxicity and Mitochondrial Oxidative Stress in the Cardiac Alterations in Diet-Induced Obesity in Rats. Cells.

[10] Feillet-Coudray C., Fouret G., Ebabe Elle R., Rieusset J., Bonafos B., Chabi B., Crouzier D., Zarkovic K., Zarkovic N., Ramos J. (2014). The mitochondrial-targeted antioxidant MitoQ ameliorates metabolic syndrome features in obesogenic diet-fed rats better than Apocynin or Allopurinol. Free Radic. Res..

[11] Coudray C., Fouret G., Lambert K., Ferreri C., Rieusset J., Blachnio-Zabielska A., Lecomte J., Ebabe Elle R., Badia E., Murphy M.P. (2016). A mitochondrial-targeted ubiquinone modulates muscle lipid profile and improves mitochondrial respiration in obesogenic diet-fed rats. Br. J. Nutr..

[12] Marin-Royo G., Rodriguez C., Le Pape A., Jurado-Lopez R., Luaces M., Antequera A., Martinez-Gonzalez J., Souza-Neto F.V., Nieto M.L., Martinez-Martinez E. (2019). The role of mitochondrial oxidative stress in the metabolic alterations in diet-induced obesity in rats. FASEB J..

[13] Chen X., Devaraj S. (2018). Gut Microbiome in Obesity, Metabolic Syndrome, and Diabetes. Curr. Diab. Rep..

[14] Dabke K., Hendrick G., Devkota S. (2019). The gut microbiome and metabolic syndrome. J. Clin. Investig..

[15] Yang T., Richards E.M., Pepine C.J., Raizada M.K. (2018). The gut microbiota and the brain-gut-kidney axis in hypertension and chronic kidney disease. Nat. Rev. Nephrol..

[16] Astudillo A.A., Mayrovitz H.N. (2021). The Gut Microbiome and Cardiovascular Disease. Cureus.

[17] Karlsson F.H., Fak F., Nookaew I., Tremaroli V., Fagerberg B., Petranovic D., Backhed F., Nielsen J. (2012). Symptomatic atherosclerosis is associated with an altered gut metagenome. Nat. Commun..

[18] Yang T., Santisteban M.M., Rodriguez V., Li E., Ahmari N., Carvajal J.M., Zadeh M., Gong M., Qi Y., Zubcevic J. (2015). Gut dysbiosis is linked to hypertension. Hypertension.

[19] Karbach S.H., Schonfelder T., Brandao I., Wilms E., Hormann N., Jackel S., Schuler R., Finger S., Knorr M., Lagrange J. (2016). Gut Microbiota Promote Angiotensin II-Induced Arterial Hypertension and Vascular Dysfunction. J. Am. Heart Assoc..

[20] Martinez B.K., White C.M. (2018). The Emerging Role of Inflammation in Cardiovascular Disease. Ann. Pharm..

[21] Saad M.J., Santos A., Prada P.O. (2016). Linking Gut Microbiota and Inflammation to Obesity and Insulin Resistance. Physiology.

[22] Cani P.D., Bibiloni R., Knauf C., Waget A., Neyrinck A.M., Delzenne N.M., Burcelin R. (2008). Changes in gut microbiota control metabolic endotoxemia-induced inflammation in high-fat diet-induced obesity and diabetes in mice. Diabetes.

[23] Ortega-Hernandez A., Martinez-Martinez E., Gomez-Gordo R., Lopez-Andres N., Fernandez-Celis A., Gutierrez-Miranda B., Nieto M.L., Alarcon T., Alba C., Gomez-Garre D. (2020). The Interaction between Mitochondrial Oxidative Stress and Gut Microbiota in the Cardiometabolic Consequences in Diet-Induced Obese Rats. Antioxidants.

[24] Blackwood E.A., Hofmann C., Santo Domingo M., Bilal A.S., Sarakki A., Stauffer W., Arrieta A., Thuerauf D.J., Kolkhorst F.W., Muller O.J. (2019). ATF6 Regulates Cardiac Hypertrophy by Transcriptional Induction of the mTORC1 Activator, Rheb. Circ. Res..

[25] Luo T., Chen B., Wang X. (2015). 4-PBA prevents pressure overload-induced myocardial hypertrophy and interstitial fibrosis by attenuating endoplasmic reticulum stress. Chem. Biol. Interact..

[26] Thuerauf D.J., Marcinko M., Gude N., Rubio M., Sussman M.A., Glembotski C.C. (2006). Activation of the unfolded protein response in infarcted mouse heart and hypoxic cultured cardiac myocytes. Circ. Res..

[27] Myoishi M., Hao H., Minamino T., Watanabe K., Nishihira K., Hatakeyama K., Aasada Y., Okada K., Ishibashi-Ueda H., Gabbiani G. (2007). Increased endoplasmic reticulum stress in atherosclerotic plaques associated with acute coronary syndrome. Circulation.

[28] Liu H., Li X., Qin F., Huang K. (2014). Selenium suppresses oxidative-stress-enhanced vascular smooth muscle cell calcification by inhibiting the activation of the PI3K/AKT and ERK signaling pathways and endoplasmic reticulum stress. J. Biol. Inorg. Chem..

[29] Kassan M., Galan M., Partyka M., Saifudeen Z., Henrion D., Trebak M., Matrougui K. (2012). Endoplasmic reticulum stress is involved in cardiac damage and vascular endothelial dysfunction in hypertensive mice. Arter. Thromb. Vasc. Biol..

[30] Li S.J., Liu C.H., Chu H.P., Mersmann H.J., Ding S.T., Chu C.H., Chen C.Y. (2017). The high-fat diet induces myocardial fibrosis in the metabolically healthy obese minipigs-The role of ER stress and oxidative stress. Clin. Nutr..

[31] Noyan-Ashraf M.H., Shikatani E.A., Schuiki I., Mukovozov I., Wu J., Li R.K., Volchuk A., Robinson L.A., Billia F., Drucker D.J. (2013). A glucagon-like peptide-1 analog reverses the molecular pathology and cardiac dysfunction of a mouse model of obesity. Circulation.

[32] Wu S., Zou M.H. (2019). Mitochondria-associated endoplasmic reticulum membranes in the heart. Arch. Biochem. Biophys..

[33] Rivera-Barahona A., Alonso-Barroso E., Perez B., Murphy M.P., Richard E., Desviat L.R. (2017). Treatment with antioxidants ameliorates oxidative damage in a mouse model of propionic acidemia. Mol. Genet. Metab..

[34] Martinez-Martinez E., Miana M., Jurado-Lopez R., Bartolome M.V., Souza Neto F.V., Salaices M., Lopez-Andres N., Cachofeiro V. (2014). The potential role of leptin in the vascular remodeling associated with obesity. Int. J. Obes..

[35] Wang M., Murdoch C.E., Brewer A.C., Ivetic A., Evans P., Shah A.M., Zhang M. (2021). Endothelial NADPH oxidase 4 protects against angiotensin II-induced cardiac fibrosis and inflammation. ESC Heart Fail..

[36] Broekmans K., Giesen J., Menges L., Koesling D., Russwurm M. (2020). Angiotensin II-Induced Cardiovascular Fibrosis Is Attenuated by NO-Sensitive Guanylyl Cyclase1. Cells.

[37] Martinez-Martinez E., Brugnolaro C., Ibarrola J., Ravassa S., Buonafine M., Lopez B., Fernandez-Celis A., Querejeta R., Satamaria E., Fernandez-Irigoyen J. (2019). CT-1 (Cardiotrophin-1)-Gal-3 (Galectin-3) Axis in Cardiac Fibrosis and Inflammation. Hypertension.

[38] Martinez-Martinez E., Buonafine M., Boukhalfa I., Ibarrola J., Fernandez-Celis A., Kolkhof P., Rossignol P., Girerd N., Mulder P., Lopez-Andres N. (2017). Aldosterone Target NGAL (Neutrophil Gelatinase-Associated Lipocalin) Is Involved in Cardiac Remodeling after Myocardial Infarction through NFkappaB Pathway. Hypertension.

[39] Ferreira J.P., Machu J.L., Girerd N., Jaisser F., Thum T., Butler J., Gonzalez A., Diez J., Heymans S., McDonald K. (2018). Rationale of the FIBROTARGETS study designed to identify novel biomarkers of myocardial fibrosis. ESC Heart Fail..

[40] Safar M.E., Czernichow S., Blacher J. (2006). Obesity, arterial stiffness, and cardiovascular risk. J. Am. Soc. Nephrol..

[41] Chen Z.W., Qian J.Y., Ma J.Y., Chang S.F., Yun J., Jin H., Sun A.J., Zou Y.Z., Ge J.B. (2014). TNF-alpha-induced cardiomyocyte apoptosis contributes to cardiac dysfunction after coronary microembolization in mini-pigs. J. Cell Mol. Med..

[42] Su Q., Li L., Sun Y., Yang H., Ye Z., Zhao J. (2018). Effects of the TLR4/Myd88/NF-kappaB Signaling Pathway on NLRP3 Inflammasome in Coronary Microembolization-Induced Myocardial Injury. Cell Physiol. Biochem..

[43] Martinez-Martinez E., Lopez-Andres N., Jurado-Lopez R., Rousseau E., Bartolome M.V., Fernandez-Celis A., Rossignol P., Islas F., Antequera A., Prieto S. (2015). Galectin-3 Participates in Cardiovascular Remodeling Associated with Obesity. Hypertension.

[44] Zhou Y., Long M.Y., Chen Z.Q., Huang J.W., Qin Z.B., Li L. (2021). Downregulation of miR-181a-5p alleviates oxidative stress and inflammation in coronary microembolization-induced myocardial damage by directly targeting XIAP. J. Geriatr. Cardiol..

[45] Orlandi M., Masi S., Bhowruth D., Leira Y., Georgiopoulos G., Yellon D., Hingorani A., Chiesa S.T., Hausenloy D.J., Deanfield J. (2021). Remote Ischemic Preconditioning Protects against Endothelial Dysfunction in a Human Model of Systemic Inflammation: A Randomized Clinical Trial. Arter. Thromb. Vasc. Biol..

[46] Marseglia L., Manti S., D’Angelo G., Nicotera A., Parisi E., Rosa G.D., Gitto E., Arrigo T. (2014). Oxidative stress in obesity: A critical component in human diseases. Int. J. Mol. Sci..

[47] McNulty M., Mahmud A., Spiers P., Feely J. (2006). Collagen type-I degradation is related to arterial stiffness in hypertensive and normotensive subjects. J. Hum. Hypertens..

[48] Johnston E.F., Gillis T.E. (2017). Transforming growth factor beta-1 (TGF-beta1) stimulates collagen synthesis in cultured rainbow trout cardiac fibroblasts. J. Exp. Biol..

[49] Duncan M.R., Frazier K.S., Abramson S., Williams S., Klapper H., Huang X., Grotendorst G.R. (1999). Connective tissue growth factor mediates transforming growth factor beta-induced collagen synthesis: Down-regulation by cAMP. FASEB J..

[50] Roy Sarkar S., Banerjee S. (2019). Gut microbiota in neurodegenerative disorders. J. Neuroimmunol..

[51] Wu S., Liu X., Jiang R., Yan X., Ling Z. (2021). Roles and Mechanisms of Gut Microbiota in Patients with Alzheimer’s Disease. Front. Aging Neurosci..

[52] Muscogiuri G., Cantone E., Cassarano S., Tuccinardi D., Barrea L., Savastano S., Colao A., on behalf of the Obesity Programs of nutrition, Education Research Assessment Group (2019). Gut microbiota: A new path to treat obesity. Int. J. Obes. Suppl..

[53] Leocadio P.C.L., Oria R.B., Crespo-Lopez M.E., Alvarez-Leite J.I. (2019). Obesity: More Than an Inflammatory, an Infectious Disease?. Front. Immunol..

[54] Boulange C.L., Neves A.L., Chilloux J., Nicholson J.K., Dumas M.E. (2016). Impact of the gut microbiota on inflammation, obesity, and metabolic disease. Genome Med..

[55] Fujii J., Homma T., Kobayashi S., Seo H.G. (2018). Mutual interaction between oxidative stress and endoplasmic reticulum stress in the pathogenesis of diseases specifically focusing on non-alcoholic fatty liver disease. World J. Biol. Chem..

[56] Bhandary B., Marahatta A., Kim H.R., Chae H.J. (2012). An involvement of oxidative stress in endoplasmic reticulum stress and its associated diseases. Int. J. Mol. Sci..

[57] Cao S.S., Kaufman R.J. (2014). Endoplasmic reticulum stress and oxidative stress in cell fate decision and human disease. Antioxid. Redox Signal..

[58] Escribano-Lopez I., Banuls C., Diaz-Morales N., Iannantuoni F., Rovira-Llopis S., Gomis R., Rocha M., Hernandez-Mijares A., Murphy M.P., Victor V.M. (2019). The Mitochondria-Targeted Antioxidant MitoQ Modulates Mitochondrial Function and Endoplasmic Reticulum Stress in Pancreatic beta Cells Exposed to Hyperglycaemia. Cell Physiol. Biochem..

[59] Fu H.Y., Okada K., Liao Y., Tsukamoto O., Isomura T., Asai M., Okuda K., Asano Y., Sanada S., Asanuma H. (2010). Ablation of C/EBP homologous protein attenuates endoplasmic reticulum-mediated apoptosis and cardiac dysfunction induced by pressure overload. Circulation.

[60] Gregor M.F., Yang L., Fabbrini E., Mohammed B.S., Eagon J.C., Hotamisligil G.H., Klein S. (2009). Endoplasmic reticulum stress is reduced in tissues of obese subjects after weight loss. Diabetes.

[61] Ozcan U., Yilmaz E., Ozcan L., Furuhashi M., Vaillancourt E., Smith R.O., Gorgun C.Z., Hotamisligil G.S. (2006). Chemical chaperones reduce ER stress and restore glucose homeostasis in a mouse model of type 2 diabetes. Science.

[62] Garcia N., Zazueta C., Aguilera-Aguirre L. (2017). Oxidative Stress and Inflammation in Cardiovascular Disease. Oxid. Med. Cell Longev..

[63] Martin R., Miana M., Jurado-Lopez R., Martinez-Martinez E., Gomez-Hurtado N., Delgado C., Bartolome M.V., San Roman J.A., Cordoba C., Lahera V. (2012). DIOL triterpenes block profibrotic effects of angiotensin II and protect from cardiac hypertrophy. PLoS ONE.

[64] Wang W., Huang X.R., Canlas E., Oka K., Truong L.D., Deng C., Bhowmick N.A., Ju W., Bottinger E.P., Lan H.Y. (2006). Essential role of Smad3 in angiotensin II-induced vascular fibrosis. Circ. Res..

[65] Cabandugama P.K., Gardner M.J., Sowers J.R. (2017). The Renin Angiotensin Aldosterone System in Obesity and Hypertension: Roles in the Cardiorenal Metabolic Syndrome. Med. Clin. N. Am..

[66] Menikdiwela K.R., Ramalingam L., Allen L., Scoggin S., Kalupahana N.S., Moustaid Moussa N. (2019). Angiotensin II Increases Endoplasmic Reticulum Stress in Adipose Tissue and Adipocytes. Sci. Rep..

[67] Ha T.S., Park H.Y., Seong S.B., Ahn H.Y. (2015). Angiotensin II induces endoplasmic reticulum stress in podocyte, which would be further augmented by PI3-kinase inhibition. Clin. Hypertens.

[68] Ramalingam L., Sopontammarak B., Menikdiwela K.R., Moustaid-Moussa N. (2020). Endoplasmic Reticulum (ER) Stress in Part Mediates Effects of Angiotensin II in Pancreatic Beta Cells. Diabetes Metab. Syndr. Obes..

[69] Malhotra J.D., Kaufman R.J. (2011). ER stress and its functional link to mitochondria: Role in cell survival and death. Cold Spring Harb. Perspect. Biol..

[70] Chen Q., Thompson J., Hu Y., Das A., Lesnefsky E.J. (2017). Metformin attenuates ER stress-induced mitochondrial dysfunction. Transl. Res..

[71] da Silva-Bertani D.C.T., Vileigas D.F., Mota G.A.F., de Souza S.L.B., Sant’Ana P.G., Freire P.P., de Tomasi L.C., Correa C.R., Padovani C.R., Fernandes T. (2020). Increased angiotensin II from adipose tissue modulates myocardial collagen I and III in obese rats. Life Sci..

