# Supplementary material for: The Interplay of Mitochondrial Oxidative Stress and Endoplasmic Reticulum Stress in Cardiovascular Fibrosis in Obese Rats
Source: Antioxidants (Basel). 2021 Aug 11;10(8):1274. doi: 10.3390/antiox10081274 (PMC8389298; doi:10.3390/antiox10081274)
Supplement: Supplementary file 1 [file antioxidants-10-01274-s001.zip › antioxidants-1303814-supplementary.pdf]

The interplay of mitochondrial oxidative stress and endoplasmic reticulum stress in cardiovascular fibrosis in obese rats

Francisco V Souza-Neto[1†], Sara Jiménez-González[1†], Beatriz Delgado-Valero[1], Raquel Jurado-López [1], Marie Genty [1], Ana Romero-Miranda [1], Cristina Rodríguez[2,3,4], María Luisa Nieto[4,5], Ernesto Martínez-Martínez [1,4,*] and Victoria Cachofeiro [1,4,*]

[1] Departamento de Fisiología, Facultad de Medicina, Universidad Complutense de Madrid–Instituto de Investigación Sanitaria Gregorio Marañón (IiSGM), Madrid, Spain. franvasc@ucm.es (FV.S-N.); saraji02@ucm.es (S. J-G.); beadel02@ucm.es (B.D-V.); rajurado@ucm.es (R.J-L.); marie.gentygau@gmail.com (M.G.); anarom12@ucm.es (A. R-M.).

[2] Institut de Recerca del Hospital de la Santa Creu i Sant Pau, Barcelona, Spain. crodriguezs@santpau.cat (C.R.).

[3] Instituto de Investigación Biomédica Sant Pau (IB Sant Pau), 08025 Barcelona, Spain.

[4] Ciber de Enfermedades Cardiovasculares (CIBERCV), Instituto de Salud Carlos III, Madrid, Spain.

[5] Instituto de Biología y Genética Molecular, CSIC-Universidad de Valladolid, Valladolid, Spain. ml.nieto@csic.es (ML.N.).

[*] These authors contributed equally to this work. Correspondence: ernmarti@ucm.es; Tel.: +34 913941483 (E.M-M.); vcara@ucm.es; Tel: +34 913941489 (V.C.).

[†] These authors contributed equally to this study.

This file includes:

1 supplemental table
6 supplemental figures

SUPPLEMENTAL TABLES

Table S1. Primers used in real time PCR analysis

Gene	Primer	Sequence (5´to 3´)
Col 1a1	Forward	GCCTCCCAGAACATCACCTA
	Reverse	ATGTCTGTCTTGCCCCAAGT
Tgf-β	Forward	CAGAAGTTGGCATGGTAGCC
	Reverse	TGCTTCAGCTCCACAGAGAA
Hprt	Forward	AGGACCTCTCGAAGTGT
	Reverse	ATTCAAATCCCTGAAGTACTCAT

Col1a1: Collagen type I; *Tgf-β*: Transforming growth factor-beta and *Hprt*: hypoxanthine-guanine phosphoribosyltransferase.

SUPPLEMENTAL FIGURES

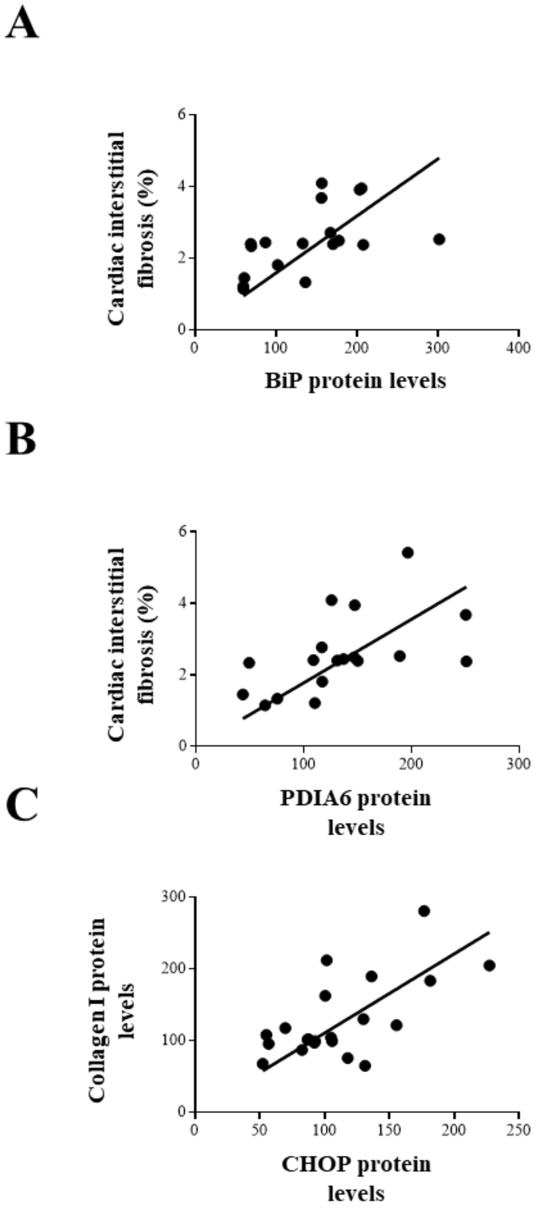

Figure S1. Correlations observed between cardiac fibrosis and endoplasmic reticulum stress. Direct correlation between cardiac interstitial fibrosis and **(A)** immunoglobulin binding protein (BiP; r=0.5475; p=0.0187); **(B)** protein disulfide isomerase family A member 6 (PDIA6; r=0.5534; p=0.0172) protein expression in all animals. **(C)** Direct correlation between cardiac CCAAT-enhancer-binding protein homologous protein (CHOP) and collagen type I protein expression in all animals (r=0.6385; p=0.0018).

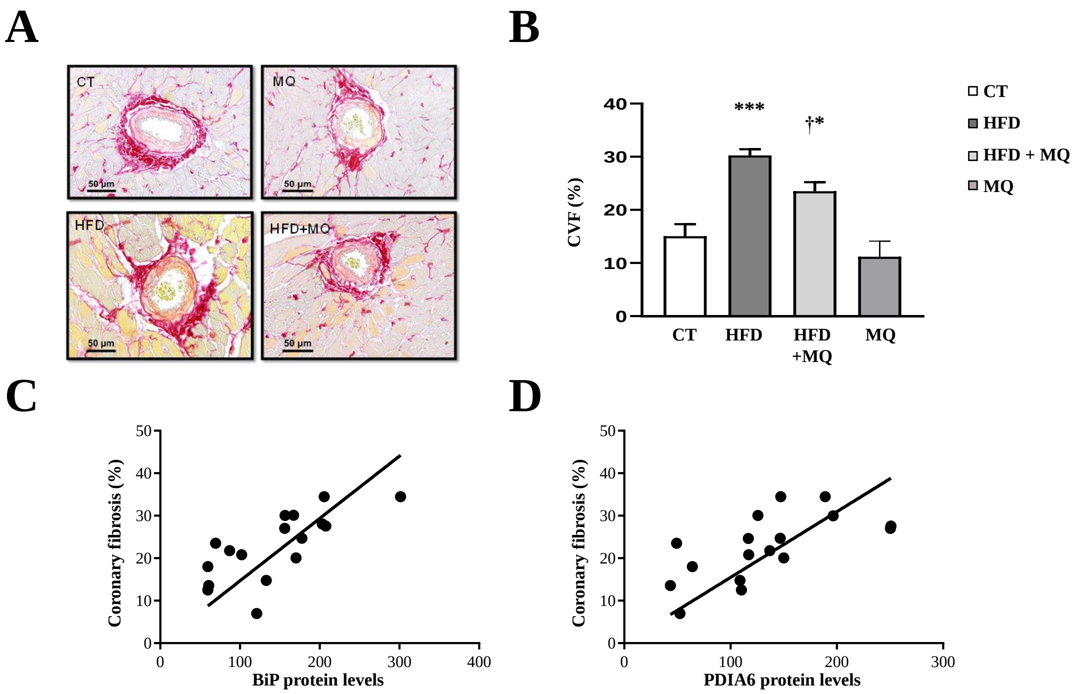

Figure S2. Mitochondrial oxidative stress mediates the fibrosis of the media of the descending coronary artery. (A) Representative microphotographs of cardiac sections staining with picrosirius red and **(B)** quantification of collagen volume fraction in the media of the coronary artery from control rats fed a normal chow (CT) and rats fed a high fat diet (HFD) treated with vehicle or with the mitochondrial antioxidant MitoQ (MQ; 200 µM). Scale bar: 50 µm. Bars graphs represent the mean ± SEM of 6-8 animals. $^{**}p<0.01$; $^{***}p<0.001$ vs. control group. †$p<0.05$ vs. HFD group. Direct correlation between coronary media fibrosis and **(C)** immunoglobin binding protein (BiP; $r=0.7207$; $p=0.0011$); **(D)** protein disulfide isomerase family A member 6 (PDIA6; $r=0.6460$; $p=0.0051$) protein expression in all animals.

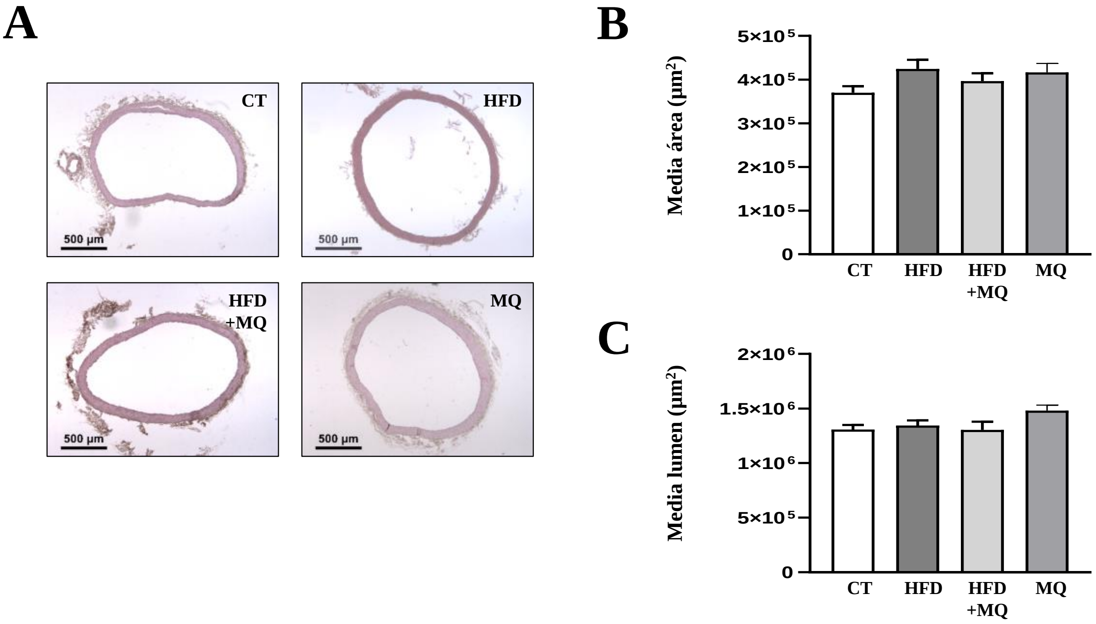

Figure S3. Vascular morphology of aorta in the animals. (A) Representative microphotographs of aortas stained with hematoxylin/eosin. **(B)** Media and **(C)** lumen area from control rats fed a normal chow (CT) and rats fed a high fat diet (HFD) treated with vehicle or with the mitochondrial antioxidant MitoQ (MQ; 200 μM). Scale bar: 500 μm. Bars graphs represent the mean ± SEM of 6-8 animals.

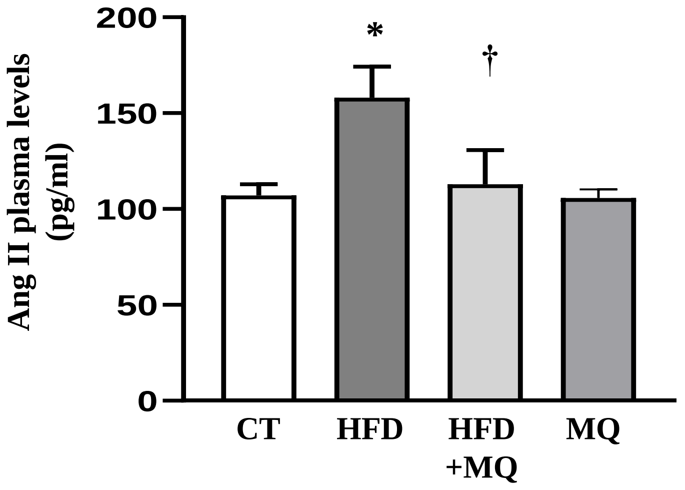

Figure S4. Angiotensin (Ang II) plasma levels from control rats fed a normal chow (CT) and rats fed a high fat diet (HFD) treated with vehicle or with the mitochondrial antioxidant MitoQ (MQ; 200 μM). Bars graphs represent the mean ± SEM of 5-8 animals. *p<0.05 vs. control group. †p<0.05 vs. HFD group.

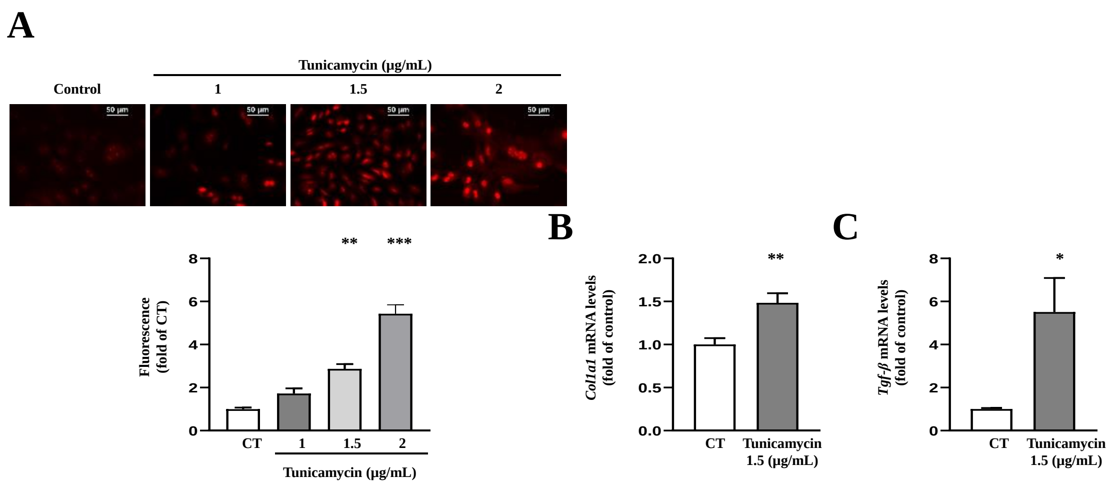

Figure S5. Endoplasmic reticulum stress increases oxidative stress and extracellular matrix markers in vascular smooth muscle cells. Effects of the endoplasmic reticulum stress inductor, tunicamycin (1-2 μg/mL) on superoxide anion production. **(A)** Representative microphotographs and quantification of cells labelled with the oxidative dye dihydroethidium (magnification 40X). mRNA levels of **(B)** collagen type I (*Col 1a1*), and **(C)** transforming growth factor-beta (*Tgf-β*) in vascular smooth muscle cells treated with tunicamycin (1.5 μg/mL)) for 24 hours. Bars graphs represent the mean ± SEM of four to six assays normalized for hypoxanthine phosphoribosyltransferase (HPRT). *p<0.05; **p<0.01, ***p<0.001 vs. control cells.

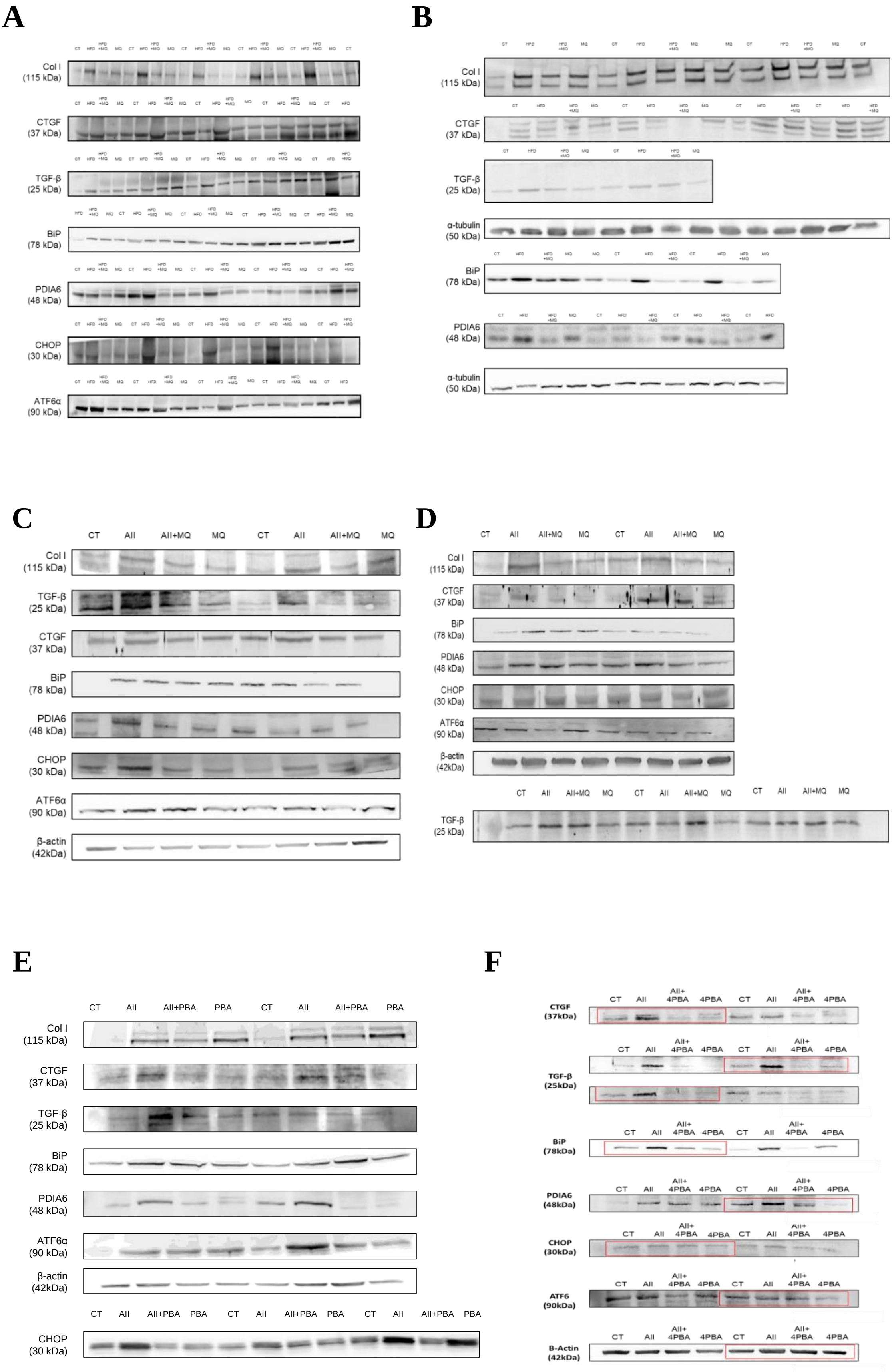

Figure S6. Original blots corresponding to (A) Figure 1; **(B)** Figure 2; **(C)** Figure 3; **(D)** Figure 4; **(E)** Figure 5 and **(F)** Figure 6.
